# Regional fat distribution and hepatic fibrosis and steatosis severity in patients with nonalcoholic fatty liver disease and type 2 diabetes

**DOI:** 10.1002/osp4.777

**Published:** 2024-07-01

**Authors:** Asieh Mansour, Saeed Pourhassan, Hadis Gerami, Mohammad Reza Mohajeri‐Tehrani, Marziye Salahshour, Ali Abbasi, Elham Madreseh, Sayed Mahmoud Sajjadi‐Jazi

**Affiliations:** ^1^ Endocrinology and Metabolism Research Center Endocrinology and Metabolism Clinical Sciences Institute Tehran University of Medical Sciences Tehran Iran; ^2^ Department of Internal Medicine Shariati Hospital Tehran University of Medical Sciences Tehran Iran; ^3^ Nutrition and Food Security Research Center Shahid Sadoughi University of Medical Sciences Yazd Iran; ^4^ Department of Cardiology Shariati Hospital Tehran University of Medical Sciences Tehran Iran; ^5^ Department of Epidemiology and Biostatistics Tehran University of Medical Sciences Tehran Iran

**Keywords:** ABSI, body fat mass, BRI, diabetes, hepatic fibrosis, hepatic steatosis, leg fat, NAFLD, trunk fat

## Abstract

**Background:**

Epidemiologic findings suggest that measures of body fat distribution predict health outcomes independent of the overall body fat assessed by body mass index (BMI). This study aimed to evaluate the associations of overall and regional body fat with the severity of hepatic steatosis and fibrosis in type 2 diabetic patients with non‐alcoholic fatty liver disease.

**Methods:**

Bioelectric impedance analysis and two newly developed anthropometric indices, namely, A Body Shape Index (ABSI) and Body Roundness Index (BRI), were used to estimate the body fat. Based on fibroscan parameters, significant hepatic fibrosis and severe steatosis were defined as ≥F2 and >66%, respectively.

**Results:**

Higher total body fat (odds ratio [OR] 1.107, 95% confidence intervals (CI) 1.038–1.182, *p* = 0.002), trunk fat (OR 1.136, 95% CI 1.034–1.248, *p* = 0.008) and leg fat (OR 1.381, 95% CI 1.139–1.674, *p* = 0.001) were associated with liver fibrosis. However, in contrast to the total body fat (OR 1.088, 95% CI 1.017–1.164, *p* = 0.014) and leg fat (OR 1.317, 95% CI 1.066–1.628, *p* = 0.011), the trunk fat was not associated with severe hepatic steatosis. BRI performed better than trunk, leg and total body fat in predicting hepatic steatosis (OR 2.186, 95% CI 1.370–3.487, *p* = 0.001) and fibrosis (OR 2.132, 95% CI 1.419–3.204, *p* < 0.001). Moreover, the trunk to leg fat ratio and ABSI were not independent predictors of either steatosis or fibrosis (*p* > 0.05).

**Conclusion:**

BRI revealed a superior predictive ability for identifying the degree of hepatic steatosis and stiffness than other obesity indices. Additionally, higher levels of adiposity in the trunk, legs, and overall body were linked to an increased risk of developing liver fibrosis. Although trunk fat did not show an association with severe hepatic steatosis, an increase in leg and total fat was related to liver steatosis.

## 1 INTRODUCTION

Non‐alcoholic fatty liver disease (NAFLD) remains the most common chronic liver disease and is one of the leading causes of morbidity, becoming a worldwide public health concern.^1^ People with diabetes have a 3‐fold increased risk of death due to NAFLD or nonalcoholic steatohepatitis (NASH).^2^ The prevalence of diabetes and NAFLD has increased significantly in the recent decades, mostly due to the worldwide epidemic of obesity.^3^

The most widely used anthropometric index for the diagnosis of excess adipose tissue (obesity) is body mass index (BMI).^4^ However, BMI is not adequate for the assessment of obese persons as it does not provide the assessment of fat distribution.^5^ Most studies have used anthropometric measures such as waist circumference, waist‐to‐hip ratio and skin fold measurements to evaluate regional fat distributions.^6^, ^7^, ^8^ In the recent years, several novel anthropometric indices have been standardized to assess both the quantity of visceral adipose tissue and body fat. Two of these indices are A Body Shape Index (ABSI) and Body Roundness Index (BRI).^9^ Meanwhile, other studies have used computed tomography (CT), dual‐energy X ray absorptiometry (DXA), or bioelectrical impedance analysis (BIA) for the measurement of body composition. DXA and CT are the gold standards considered for adiposity distribution assessment, but their use is limited due to radiation exposure and less availability. Compared to anthropometric methods, BIA has the advantage of measuring both total body and regional adiposity, which is similar to DXA and CT.^10^, ^11^, ^12^

Several studies have reported a positive association between visceral adipose tissue and chronic diseases, in particular cardiovascular diseases (CVD), type 2 diabetes mellitus (T2DM)^13^ and NAFLD.^14^, ^15^ Recent reports using DXA to assess visceral adipose tissue have also shown that the visceral adipose tissue has independent and positive associations with the severity of hepatic steatosis and liver stiffness in patients with and without T2DM.^16^ Chen et al. demonstrated that individuals with a greater central body fat had a higher risk of hepatic steatosis using a large population‐based study.^17^ In addition, Jung et al. investigated the cross‐sectional association between visceral‐to‐subcutaneous fat ratio and the risk of NAFLD in 7465 Korean adults who participated in a health screening examination. They showed that an increased visceral‐to‐subcutaneous fat ratio was significantly associated with a higher risk of NAFLD and advanced fibrosis.^18^ Some studies have also suggested that lower leg fat mass is a risk factor for the development of metabolic diseases^19^ including NAFLD.^20^

Previous research has highlighted the unique impact of visceral fat on metabolic disorders and NAFLD.^21^ Nevertheless, investigations into the relationship between leg fat mass, other regional adipose tissue distributions, novel anthropometric measures, and NAFLD risk have been sparse and inconclusive.^22^

In addition, there is not much evidence regarding the effect of regional body fat distribution on the severity of hepatic steatosis and fibrosis among patients with concurrent diabetes and NAFLD. Therefore, the impact of adiposity distribution in these patients needs to be further explored.

Thus, the present study aimed to investigate the association between obesity indexes, including trunk fat mass, leg fat mass and total fat mass using BIA as well as BRI and ABSI and the severity of hepatic steatosis and fibrosis in T2DM patients with NAFLD.

## 2 METHODS

### 2.1 Study design

This cross‐sectional study was a secondary analysis of the baseline data from a randomized controlled trial (RCT) in the patients with diabetes who had a primary diagnosis of NAFLD. RCT was prospectively performed at the Endocrinology and Metabolism Research Center, Tehran University of Medical Sciences, Tehran, Iran. The protocol of the study is presented at the Iranian Registry of Clinical Trials (irct.ir) with the trial registration number IRCT201707024010N21.

T2DM patients were eligible for inclusion if their age was 30–53 years, had a controlled attenuation parameter score ≥260 dB/m, BMI <40 kg/m^2^ and had not taken insulin at the time of study. Exclusion criteria were as follows: abnormal viral hepatitis test, a known history of severe liver or renal disease, clinically significant cardiovascular conditions, malignant neoplasms, or a history of any type of mental illness.

### 2.2 Anthropometry and body composition

Height was measured using a stadiometer to the nearest 0.1 cm without shoes. BIA (BC 418 MA Segmental Body Composition Analyzer) was used to measure body weight, total body fat, leg fat and trunk fat mass. BMI was calculated as weight in kilograms divided by square of height in meters. The following equations were used to calculate BRI and ABSI.^23^, ^24^

(1) $$\text{BRI}=364.2-365.5\sqrt{1-\left(\frac{{\left(\frac{\text{WC}}{\left(2\pi \right)}\right)}^{2}}{{\left(0.5\times \text{height}\right)}^{2}}\right)}$$

(2) $$\text{ABSI}=\frac{\text{WC}}{\left(\text{BM}I^{\frac{2}{3}}\times \text{heigh}t^{\frac{1}{2}}\right)}$$

### 2.3 Liver steatosis and fibrosis

Liver stiffness and steatosis measurements were performed using Fibroscans 502 instrument (EchoSense, 5 MHz), a medical device based on transient elastography. The detailed protocol of the measurement and calculations has been described previously.^25^ Hepatic steatosis was categorized as follows: *S*0 = 5%, *S*1 = 5%–33%, *S*2 = 34%–66%, and *S*3 >66%; steatosis higher than 66% was defined as severe steatosis. Hepatic fibrosis was categorized according to the METAVIR scoring system as follows: F0, F1, F2, F3 and F4; *F* ≥ 2 was considered as significant fibrosis.^26^

### 2.4 Laboratory parameter

From 8:00 to 9:00 AM, a blood sample was drawn from each patient to perform the following laboratory investigations: Alanine transaminase (ALT), aspartate aminotransferase (AST), gamma‐glutamyl transferase (GGT), and lipids, which were performed using an enzyme‐linked immunosorbent assay (ELISA) kit (Roche, Germany). The concentration of hemoglobin A1c (HbA1c) was assessed using a high performance liquid chromatography analyzer (Tosoh, Tokyo, Japan).

### 2.5 Hypertension

Systolic blood pressure (SBP) and diastolic blood pressure (DBP) values were measured with a mercury sphygmomanometer after at least a 10‐min rest. Participants were considered hypertensive based on SBP ≥130 mm Hg and/or DBP ≥80 mm Hg or taking anti‐hypertensive medications.^27^

Ethics approval was obtained from the Ethics Committee of Tehran University of Medical Sciences (IR.TUMS.DDRI.REC.1400.045) and the written informed consent was provided by all participants.

### 2.6 Statistical analysis

All statistical analyses were conducted using SPSS software version 24.0 (SPSS, Chicago, IL, USA). The continuous variables were presented as mean ± standard error (SE) and checked for normality using Kolmogorov‐Smirnov test. The significance of differences between the two mean values was assessed by the Mann–Whitney *U* test or independent sample *t* test for non‐normally and normally distributed data, respectively. The Chi‐square test or Fisher's exact test was used for categorical variables as deemed appropriate. Multivariable regression analysis was also conducted to examine the association between total fat mass, leg fat mass, trunk fat mass, trunk to leg fat mass ratio, ABSI and BRI (independent variable) and the severity of hepatic steatosis and fibrosis (dependent variable) in different models; model 1: unadjusted, model 2: adjusted for age and sex and model 3: adjusted for age, sex, HbA1c, physical activity, smoking, total cholesterol, high‐density lipoprotein cholesterol and triglycerides. As the original ABSI values had a small range (<0.1), we multiplied the ABSI by 100 to obtain more intuitive results. A *p*‐value <0.05 was defined as statistically significant.

## 3 RESULTS

From September 2017 to March 2018, 108 participants with T2DM and NAFLD were included. The study population consisted of 75 males and 33 females (25 premenopausal and 8 postmenopausal women). Baseline characteristics of the participants and their use of ongoing medication have been summarized according to sex in Table 1. The mean ± SE age of the participants was 44.28 ± 0.66 years for males and 45.63 ± 0.88 for females (*p* = 0.347); there were more smokers in males than in females (*p* = 0.005). There were no significant differences in hypertension history and physical activity between men and women (*p* > 0.05). Despite the non‐significant difference in the BMI (*p* = 0.465), ABSI (0.077) and trunk fat mass (*p* = 0.461) between females and males, other body composition parameters showed a significant difference between the two groups. In the female group, the total fat mass (32.21 ± 1.18 vs. 25.01 ± 0.97, *p* < 0.001) and leg fat mass (12.10 ± 0.40 vs. 7.65 ± 0.33, *p* < 0.001) were greater than those of their male counterparts. However, the trunk to leg fat mass ratio (2.07 ± 0.07 vs. 1.38 ± 0.06, *p* < 0.001) was greater in the male participants. In addition, laboratory parameters including AST, ALT, GGT and triglyceride were significantly lower, while HDL was significantly higher in female than male participants. Further, there was no meaningful imbalance in the use of ongoing medications (Table 1).

**TABLE 1:** Baseline characteristics of patients according to the sex.

Parameters^a^	Male (*n* = 75)	Female (*n* = 33)	*p*‐value
Age (year)^b^	44.28 ± 0.66	45.63 ± 0.88	0.347
Smoking (yes)^c^	22 (29.30)	2 (6.10)	0.005
Physical activity (METs h/day)^d^	31.10 ± 0.60	32.34 ± 0.60	0.146
Hypertension (yes)^c^	64 (85.30)	27 (81.80)	0.644
BMI (kg/m^2^)^d^	30.54 ± 0.47	31.13 ± 0.54	0.465
Total fat mass (kg)^b^	25.01 ± 0.97	32.21 ± 1.18	<0.001
Leg fat mass (kg)^d^	7.65 ± 0.33	12.10 ± 0.40	<0.001
Trunk fat mass (kg)^d^	15.62 ± 0.77	16.56 ± 0.77	0.461
Trunk fat/leg fat mass ratio^d^	2.07 ± 0.07	1.38 ± 0.06	<0.001
BRI^b^	6.10 ± 0.17	6.68 ± 0.25	0.026
ABSI^d^	0.085 ± 0.0003	0.083 ± 0.0008	0.077
Sever liver steatosis (>66% or S3)^e^	25 (33.00)	7 (21.20)	0.807
Fibrosis (≥F2)^e^	25 (78.10)	7 (21.90)	0.204
ALT (U/L)^b^	24.50 ± 1.14	18.09 ± 1.94	<0.001
AST (U/L)^b^	27.13 ± 1.13	24.06 ± 2.17	0.010
GGT (U/L)^b^	47.39 ± 4.22	30.27 ± 2.41	<0.001
HbA1c (%)^d^	8.48 ± 0.53	7.97 ± 0.28	0.511
Total cholesterol (mg/dL)^d^	164.89 ± 4.13	169.48 ± 6.93	0.554
Triglycerides (mg/dL)^b^	234.49 ± 25.90	162.54 ± 13.15	0.011
HDL‐C (mg/dL)^d^	32.45 ± 0.95	41.06 ± 1.35	<0.001
Medications (yes)^e^			
Metformin	70 (93.30)	31 (93.90)	0.906
DPP4 Inhibitors	23 (30.70)	10 (30.30)	0.970
Sulfonylureas	28 (37.30)	9 (27.30)	0.310
Thiazolidinediones	4 (5.30)	1 (3.00)	0.600
Aspirin	10 (13.30)	3 (9.10)	0.533
Lipid‐lowering medications	29 (38.70)	13 (39.40)	0.943
Anti‐hypertensive medications (beta‐blockers, ACE inhibitors, ARBS)	11 (14.70)	10 (30.30)	0.059

Abbreviations: ABSI, a body shape index; ACE, angiotensin‐converting enzyme; ALT, alanine aminotransferase; ARBS, angiotensin II receptor blockers; AST, aspartate aminotransferase; BRI, body roundness index; DPP4, dipeptidyl peptidase 4; GGT, gamma‐glutamyl transferase; HbA1c, hemoglobinA1c; HDL‐C, high‐density lipoprotein cholesterol; METs, metabolic equivalents.

^a^ Values are expressed as mean ± standard error or number (%).

^b^ Mann‐Whitney *U* test.

^c^ Fisher's exact test.

^d^ Independent sample *t* test

^e^ Chi‐squared test.

Multivariate logistic regression analysis showed that the total fat mass was associated with severe hepatic steatosis (odds ratio [OR] 1.088, 95% confidence intervals (CI) 1.017–1.164, *p* = 0.014) and fibrosis (OR 1.107, 95% CI 1.038–1.182, *p* = 0.002). With regard to regional fat distribution, leg fat mass was associated with both severe hepatic steatosis (OR 1.317, 95% CI 1.066–1.628, *p* = 0.011) and fibrosis (OR 1.381, 95% CI 1.139–1.674, *p* = 0.001), but trunk fat mass was only associated with liver fibrosis (OR 1.136, 95% CI 1.034–1.248, *p* = 0.008). Meanwhile, the trunk to leg fat ratio and ASBI were not associated with either steatosis or fibrosis (Table 2). On the other hand, BRI performed better than other obesity indexes in predicting hepatic steatosis and fibrosis; a unit increase in BRI was associated with a 2.18‐ fold increase in the odds of hepatic steatosis (OR 2.186, 95% CI 1.370–3.487, *p* = 0.001) and a unit increase in BRI was associated with a 2.13‐ fold increase in the odds of liver fibrosis (OR 2.132, 95% CI 1.419–3.204, *p* < 0.001).

**TABLE 2:** Association of body composition with severity of hepatic steatosis and fibrosis among patients with NAFLD and T2DM (*n* = 108).

Variable	Liver steatosis (>66%)			Fibrosis (≥F2)		
	OR	0.95% CI	*p*‐value	OR	0.95% CI	*p*‐value
Leg fat mass (Kg)						
Model 1 (unadjusted)	1.156	1.007–1.326	0.039	1.148	1.012–1.303	0.031
Model 2	1.327	1.080–1.632	0.007	1.359	1.130–1.636	0.001
Model 3	1.317	1.066–1.628	0.011	1.381	1.139–1.674	0.001
Trunk fat mass (Kg)						
Model 1 (unadjusted)	1.062	0.977–1.153	0.156	1.106	1.020–1.198	0.014
Model 2	1.063	0.977–1.157	0.155	1.112	1.021–1.212	0.015
Model 3	1.071	0.982–1.168	0.123	1.136	1.034–1.248	0.008
Total fat mass (Kg)						
Model 1 (unadjusted)	1.062	1.006–1.121	0.029	1.070	1.015–1.127	0.011
Model 2	1.083	1.016–1.153	0.014	1.103	1.036–1.175	0.002
Model 3	1.088	1.017–1.164	0.014	1.107	1.038–1.182	0.002
Trunk fat to leg fat mass ratio						
Model 1 (unadjusted)	0.729	0.375–1.417	0.351	1.043	0.533–2.043	0.902
Model 2	0.628	0.281–1.401	0.256	0.795	0.324–1.948	0.615
Model 3	0.719	0.316–1.632	0.430	0.919	0.341–2.476	0.867
ABSI*100						
Model 1 (unadjusted)	2.943	0.909–9.526	0.072	2.962	0.894–9.813	0.076
Model 2	3.195	0.904–11.293	0.071	2.511	0.708–8.909	0.154
Model 3	3.299	0.892–12.196	0.074	4.159	0.928–18.648	0.063
BRI						
Model 1 (unadjusted)	1.875	1.272–2.764	0.001	1.861	1.314–2.636	<0.001
Model 2	2.069	1.354–3.161	0.001	2.089	1.403–3.110	<0.001
Model 3	2.186	1.370–3.487	0.001	2.132	1.419–3.204	<0.001

*Note*: Model 2 includes variables from model 1 plus age and sex; Model 3 includes variables from model 2 plus HbA1c, physical activity, smoking, total cholesterol, HDL‐C, and triglycerides.

Abbreviations: ABSI, a body shape index; BRI, body roundness index.

## 4 DISCUSSION

A number of different methods, which vary in their accuracy and feasibility, are commonly used to identify who are overweight or obese.^28^ In the present study, we used BIA and two new obesity indexes, BRI and ABSI, to investigate the relationship between regional adiposity and the severity of steatosis as well as fibrosis among T2DM patients with NAFLD. In this study, trunk fat mass and leg fat mass were both predictors of fibrosis. Interestingly, leg fat mass, not trunk fat mass, was associated with severe steatosis. In contrast, the ratio of trunk to leg fat mass showed no significant association with either fibrosis or severe steatosis. Current results also indicated that the total fat mass and BRI could be independent parameters for severe steatosis and fibrosis.

A positive relationship between trunk fat mass or waist circumference and the risk of NAFLD was confirmed.^29^, ^30^, ^31^ In the same way, this study showed that individuals with increased trunk fat mass were at a higher risk for fibrosis. However, we did not find an association between trunk fat mass and severe hepatic steatosis in the patients with T2DM and NAFLD. This discrepancy with the previous studies^32^, ^33^ could be explained by the fact that during the progression of fibrosis or cirrhosis, steatosis may disappear; this condition is also called burnt‐out non‐alcoholic steatohepatitis (NASH).^34^

In this study, both leg fat and total fat mass were associated with severe hepatic steatosis and fibrosis. Previous studies have found the opposite association between leg fat and liver enzyme levels,^35^ hepatic steatosis^17^ and also, NAFLD risk,^20^ especially in women.^36^ These studies suggest that the observed effects may be mediated by the differences in lipolytic activity and/or secretion of bioactive peptides in the subcutaneous leg fat in comparison to central visceral fat mass; leg adipose tissue is more likely to take up non‐esteriﬁed fatty acid, which could prevent excess fat accumulation in the liver.^20^, ^36^ The data obtained from 14,502 individuals who participated in the Korea National Health and Nutrition Examination Survey during the 2008–2011 period also showed an inverse association between leg fat to total fat ratio and NAFLD risk using the logistic regression analysis. Additionally, a lower leg fat/total fat ratio was associated with the increased risks of advanced liver fibrosis among subjects with NAFLD.^20^

In that study, hepatic steatosis and fibrosis were defined by clinical criteria only and not by imaging.^20^ Therefore, the methods used to define hepatic steatosis and fibrosis might be one of the reasons for the observed variation.

However, all studies have not shown consistent results. For example, a cross‐sectional analysis conducted in China reported that an increase in calf circumference was significantly associated with NAFLD and insulin resistance. The association of calf circumference with insulin resistance was stronger in subjects whose BMI ≥24 kg/m2, as compared to normal weight subjects.^22^ All participants of this study were diabetic patients and had BMI ≥24 kg/m2 except one, who had BMI = 23.

In addition, there was a sex difference in the relationship between body fat distribution and NAFLD risk.^36^, ^37^ Jun et al. showed that in 408 patients without diabetes, femoral subcutaneous fat amounts were inversely related to NAFLD in women but not in men. In that study, ultrasound scan and CT were used for measuring fatty liver and adiposity, respectively.^36^ Additionally, in women with NAFLD, menopause may have an additional independent effect. Suzuki et al. indicated that the accumulation of fat in the extremities was associated with more severe liver fibrosis in premenopausal women.^38^ In this dataset, the study population mainly consisted of men (male to female ratio was approximately 2:1) and only eight participants were postmenopausal. Therefore, it was not possible to examine the association between sex and menopausal status and the severity of steatosis or fibrosis.

Differences in the choice of the techniques used for measuring fat distribution and diagnosing NAFLD or the study population could explain some of these discrepancies. Another interpretation of the present results is that these observations could primarily reflect an approximate linear relationship between leg fat and total fat mass. In this study, total fat mass was strongly correlated with leg fat mass (*r* = 0.90, *p* < 0.001) and trunk fat mass (*r* = 0.82, *p* < 0.001) (data not shown). According to the previous epidemiologic, cross‐sectional, and longitudinal studies, a high amount of total fat is a well‐established risk factor for NAFLD.^39^, ^40^, ^41^

A cross‐sectional study in the Netherlands, which focused on participants aged 45 years or older, found that high fat mass was significantly correlated with a higher risk of NAFLD in both normal weight and overweight individuals.^42^ Besides, a community‐based study of 3419 Chinese adults suggested that the high fat mass/fat free mass ratio was associated with higher NAFLD and fibrosis risk and this ratio could be considered as a novel predictor of the development of NAFLD and fibrosis in both obese and non‐obese participants.^43^ As individuals with a higher total fat mass tend to have more fat in their legs or trunk, the associations between the leg fat mass and the risk of NAFLD severity may be primarily related to the increased amount of total fat mass. Thus, overall fatness rather than fat mass distribution may modulate NAFLD severity. Current findings may, thus, suggest that interventions that predominantly reduce total fat mass, rather than leg or trunk fat mass, may be more beneficial in NAFLD improvement.

Finally, in this study, compared to ABSI and conventional anthropometric indices including total fat mass, trunk fat mass, and leg fat mass, BRI showed a higher predictive ability in diagnosing liver fibrosis and steatosis. BRI was designed by Thomas et al.^23^ to determine the percentage of body fat as well as the percentage of visceral adipose tissue using measurements of waist circumference and height. Several observational studies have indicated that the BRI could be a valuable tool in identifying NAFLD, showing greater diagnostic precision than BMI in predicting NAFLD^44^ and metabolic syndrome.^45^ However, a comprehensive meta‐analysis is needed to establish whether BRI outperforms BMI and other obesity indices as a predictor of NAFLD.

This study faced several limitations. Since this study had a cross‐sectional design, a causal relationship between the regional body fat mass and the severity of liver steatosis or fibrosis could not be drawn. The small sample size was another limitation of the present study. In addition, despite the conclusions that could be derived from multivariate regression analysis with adjustment for potential confounders, additional large‐scale studies with longer follow‐up periods are necessary to confirm the reported links between regional body fat mass and the severity of hepatic steatosis or fibrosis. Finally, because of the selection of T2DM patients, the results of the current study cannot be applied to all NAFLD patients.

## 5 CONCLUSION

These results suggest that in T2DM patients with NAFLD, a higher amount of leg fat mass could be as important as a higher amount of trunk fat mass in determining the severity of NAFLD. In addition, BRI may serve as a reliable indicator for detecting NAFLD, surpassing traditional anthropometric indices. However, there are still some questions which should be addressed by further studies. Are different patterns of fat distribution more closely related to NAFLD severity than total fat mass? Is the impact of adipose distribution on the severity of NAFLD the same in females and males?

## AUTHOR CONTRIBUTIONS

**Asieh Mansour**: Study conception and design, acquisition of data, analysis and interpretation of data, Drafting of manuscript, Critical revision. **Saeed Pourhassan**: Critical revision. **Hadis Gerami**: Acquisition of data. **Mohammad Reza Mohajeri‐Tehrani**: Critical revision. **Marziye Salahshour**: Acquisition of data. **Ali Abbasi**: Critical revision. **Elham Madreseh**: Analysis and interpretation of data. **Sayed Mahmoud Sajjadi‐Jazi**: Study conception and design, Analysis and interpretation of data, Drafting of manuscript, Critical revision. All authors have read the manuscript and approved the manuscript.

## CONFLICT OF INTEREST STATEMENT

None of the authors have any conflicts of interest or financial ties to disclose.

## Data Availability

Data are available upon reasonable request. To access the required data, the researchers can contact Dr. Asieh Mansour, Email: asiehmansour@yahoo.com.
